# CD155 knockdown promotes apoptosis *via* AKT/Bcl‐2/Bax in colon cancer cells

**DOI:** 10.1111/jcmm.13301

**Published:** 2017-08-16

**Authors:** Qianqian Zheng, Biao Wang, Jian Gao, Na Xin, Wei Wang, Xiaowen Song, Yue Shao, Chenghai Zhao

**Affiliations:** ^1^ Department of Pathophysiology College of Basic Medical Science China Medical University Shenyang China; ^2^ Department of Biochemistry and Molecular Biology College of Basic Medical Science China Medical University Shenyang China; ^3^ Center of Laboratory Technology and Experimental Medicine China Medical University Shenyang China

**Keywords:** colorectal cancer, CD155, apoptosis, proliferation

## Abstract

CD155, one of the nectin‐like molecule family members, is involved in cell adhesion and motility. CD155 is overexpressed in several human cancers, but its role in proliferation and apoptosis of colorectal cancer remains unclear. We found that CD155 was up‐regulated in colorectal cancer tissues. CD155 knockdown *via* shRNA lentiviruses inhibited colon cancers cell migration and invasion, with a reduction in the expression of FAK, Src and MMP‐2. CD155 down‐regulation also suppressed colon cancer cell proliferation, accompanied by changing expressions of some molecules related to cell cycle. Finally, CD155 knockdown increased the expression ratio between Bax and Bcl‐2, resulting in a significant increase in colon cancer cell apoptosis. Taken together, these results demonstrate that CD155 is involved in not only migration and invasion but also proliferation and survival abilities of colon cancer cells, suggesting that CD155 is one of key molecules promoting the growth and metastasis of colorectal cancer.

## Introduction

CD155 was originally identified as the receptor of poliovirus, a membrane protein with conserved amino acids and domain structure characteristic similar to the immunoglobulin superfamily 1. CD155 is also called Necl‐5, belonging to the nectin‐like molecule family. These immunoglobulin‐like molecules harbour domain structures that are similar to those of nectins. It was demonstrated that Necl‐5 interacted with nectin3 in cell–cell junctions 2. Interestingly, this interaction down‐regulated Necl‐5 by endocytosis, which inhibited the movement of NIH3T3 cells 3. On the other side, overexpression of Necl‐5 in cell surface suppressed contact inhibition and increased cell motility 4.

CD155 can regulate the functions of some growth factors. It enhanced the platelet‐derived growth factor (PDGF)‐induced activation of the Ras‐Raf‐MEK‐ERK signalling and proliferation of NIH3T3 cells 5. Moreover, fibroblast growth factor (FGF) induced CD155 expression through the Raf‐MEK‐ERK‐AP‐1 pathway in NIH3T3 cells 6. CD155 loss in human umbilical vein endothelial cells (HUVECs) impaired VEGF‐induced capillary‐like network formation, migration and proliferation, but promoted apoptosis 7.

Several types of human malignancies such as lung adenocarcinoma, melanoma, pancreatic cancer and colon cancer have been shown to overexpress CD155 8, 9, 10, 11. As supposed to be, CD155 expression was correlated with tumour progression and unfavourable prognosis 8, 9, 10. CD155 knockdown inhibited migration and invasion of glioblastoma and glioma cells 12, 13. Some mechanisms underlying CD155‐induced metastasis have been explored. It was reported that CD155 could activate Src/focal adhesion kinase (FAK) signalling 13. Moreover, CD155 knockdown suppressed proliferation of pancreatic cancer cells and ras‐mutated cells 10, 14.

CD155 is overexpressed in colorectal cancer tissues; however, the role of CD155 in colon cancer remains unknown. We asked whether CD155 is involved in colon cancer cell proliferation, invasion and migration by transfecting mouse colon cancer cell CT26 and human colon cancer cell Sw620 with CD155 shRNA. As CD155 is related to the function of some growth factors, we also investigated the involvement of CD155 in colon cancer cell apoptosis. Our results demonstrated that CD155 knockdown not only inhibited migration, invasion and proliferation, but also induced apoptosis of colon cancer cells. Consistently, our *in vivo* study indicated that CD155 knockdown impeded tumour growth.

## Materials and methods

### Patients and tissue specimens

Paraffin‐embedded tissues were obtained from the Department of Pathology, China Medical University. None of the patients had previously undergone radiotherapy or chemotherapy or any other therapy before the surgery. The main characteristics of the individuals enrolled are shown in Table 1. Clinical and pathological reports were reviewed for age, gender, tumour location, Duke's Degree, differentiation and AJCC stage. Colorectal cancers were graded according to the World Health Organization (WHO) classification. Approval was obtained for the use of clinical materials for research purposes by the Institutional Research Ethics Committee of China Medical University. All samples were collected and analysed with the prior written informed consent of the patients.

**Table 1:** Correlation between CD155 expression and clinicopathological characteristics

	Total (*n* = 97)	CD155 protein expression		*P*‐value
		Negative (*n* = 14)	Positive (*n* = 83)	
Age (%)				
<65	44 (45.4)	8 (57.1)	36 (43.4)	0.393
≥65	53 (54.6)	6 (42.9)	47 (56.6)	
Gender (%)				
Male	57 (58.8)	9 (64.3)	48 (57.8)	0.773
Female	40 (41.2)	5 (35.7)	35 (42.2)	
Tumour location (%)				
Right	24 (24.7)	3 (21.4)	21 (25.3)	0.955
Transverse	10 (10.3)	2 (14.3)	8 (9.6)	
Left	35 (36.1)	5 (35.7)	30 (36.2)	
Rectum	28 (28.9)	4 (28.6)	24 (28.9)	
Duke's Degree (%)				
A	10 (10.3)	5 (35.7)	5 (6.0)	0.008
B	38 (39.2)	5 (35.7)	33 (39.8)	
C	27 (27.8)	2 (14.3)	25 (30.1)	
D	22 (22.7)	2 (14.3)	20 (24.1)	
Differentiation (%)				
Well	31 (32.0)	4 (28.6)	27 (32.5)	0.53
Moderate	36 (37.1)	7 (50.0)	29 (35.0)	
Poor	30 (30.9)	3 (21.4)	27 (32.5)	
AJCC stage (%)				
I + II	42 (43.3)	11 (78.6)	31 (37.3)	0.007
III + IV	55 (56.7)	3 (21.4)	52 (62.7)	
Metastasis (%)				
No	37 (38.1)	12 (85.7)	35 (42.2)	0.003
Yes	60 (61.9)	2 (14.3)	48 (57.8)	

*P* < 0.05 indicates a significant association between the variables.

Statistical analysis was carried out with Pearson chi‐square test.

### Antibodies

The rabbit anti‐CD155 polyclonal antibody (ab103630) was obtained from Abcam, Cambridge, UK. The cell cycle regulation antibody sampler kit (#9932), the rabbit anti‐FAK polyclonal antibody (#3285), the rabbit anti‐MMP‐2 polyclonal antibody (#4022), the rabbit anti‐Src polyclonal antibody (#2123), the rabbit anti‐Akt polyclonal antibody (#9272), the rabbit anti‐phospho‐Akt (Ser473) polyclonal antibody (#9271), the rabbit anti‐Bcl‐2 monoclonal antibody (#3498), the rabbit anti‐Bax polyclonal antibody (#2772), the apoptosis antibody sampler kit (#9915), the anti‐rabbit IgG HRP‐linked antibody (#7074) and antimouse IgG HRP‐linked antibody (#7076) were obtained/purchased from Cell Signaling Technology (CST), Boston, USA.

### Immunohistochemistry

Briefly, the tissue sections were consecutively deparaffinized in xylene (I, II and III) and rehydrated in a graded alcohol series (100% alcohol, 95% alcohol, 85% alcohol and 75% alcohol). Antigen retrieval process was performed in 0.01 M sodium citrate solution (pH 6.0) in a high‐pressure steam boiler for 10 min. Non‐specific binding was blocked by incubating the sections in phosphate‐buffered saline supplemented with 10% normal goat serum at room temperature for 1 hr. Immunoreactivity was evaluated separately by two experienced pathologists who were blinded to the clinicopathological data of the participants.

### Image analysis

Immunolabelled and sampled tumour sections were observed using a Leica DMRB microscope (20× and 40× magnification) (Leica, Wetzlar, Germany). Five randomly chosen fields of view were assessed. We used a staining index (SI; values 0–12) with the following formula: SI = staining intensity × staining area, where intensities were scored semiquantitatively as follows: 0 (negative staining), 1 (mild staining), 2 (moderate staining) and 3 (intense staining). The staining area was scored as follows: 0, no staining of cells; 1, 1–25%; 2, 26–50%; 3, 51–75%; and 4, 76–100%. SI was graded as follows: 0–1, negative expression; 2–4, weakly positive expression; 5–7, moderately positive expression and 8–10, strongly positive expression. All of the three levels were considered as positive.

### Immunofluorescence (IF) histochemistry and confocal imaging

Sections were permeabilized with 0.3% Triton X‐100 diluted in PBS for 20 min. and then blocked with 10% normal goat serum for 1 hr at room temperature followed by incubation with specific primary antibodies at 4°C overnight. After washed with PBS three times for 5 min. each time, the slides were subjected to fluorescent secondary antibody for 1 hr at room temperature. DAPI was used to detect the nuclei as counter staining. All samples were imaged on confocal microscope (FV‐1000; Olympus, Tokyo, Japan).

### Cell culture

The human CRC cell lines HT29, Caco2, Sw480, Sw620, HCT116 and LoVo were cultured in RPMI 1640 medium (Gibco, New York, USA) with 10% foetal bovine serum (FBS) (Biological Industries, Israel). CT26 cells, a colon adenocarcinoma cell line, were maintained in Dulbecco's modified Eagle's medium (DMEM). Cells were cultured at 37°C in a humidified incubator with 5% CO_2_. The cells were digested using 0.25% trypsin and 0.02 mol/l EDTA in PBS and seeded in 6‐ and 12‐well plates for the experiments.

### Plasmids construction and lentiviral transduction

The control short hairpin RNA (shRNA) lentivirus (scramble) and CD155 shRNA lentivirus (siCD155‐565, siCD155‐566 and siCD155‐567) were all constructed by Obio Technology Corp., Ltd (Shanghai, China). The shRNA sequences specifically targeting CD155 (siCD155‐565 Target: 5′‐CCGTAGAGGATGGTCTCAA‐3′; siCD155‐566 Target: 5′‐GGGCCAAGTGCACATCATT‐3′; siCD155‐567 Target: 5′‐CCTAGGCTACATCTTTCTT‐3′) and Scramble Target: 5′‐TTCTCCGAACGTGTCACGT‐3′ were cloned into pLKD‐CMV‐Puro‐U6‐shRNA vector. For lentivirus transfections, CT26 and Sw620 cells were transfected with 25 MOI of scramble or CD155 knockdown lentivirus.

### RNA isolation and quantitative real‐time PCR (qPCR)

Total RNA was extracted from tissues using TRIzol™ reagent (TaKaRa, Dalian, China) according to the manufacturer's instructions. Reverse transcription was carried out in a 25 μl reaction volume with 2 μg of total RNA according to the manufacturer's protocol for M‐MLV reverse transcriptase (Promega, Madison, WI, USA). The qPCR primers were provided by Genscript Company. The sequences of forward and reverse primers are shown in Table 2. Relative quantities (Δ cycle threshold (*C*_t_) value) were obtained by subtracting the *C*_t_ value of CD155 from 18 sec. The fold change was calculated according to the formula 2^−ΔΔCt^. Each reaction was performed in triplicate.

**Table 2:** The sequences of forward and reverse primers

mRNA	Sequence
CD155	
Forward	5‐GCTAGAAGGACTCACTAGACTCAGGAA‐3
Reverse	5‐GTCGCCTCATCTGTCGTGGAAC‐3
18S	
Forward	5‐GCAGAATCCACGCCAGTACAAGAT‐3
Reverse	5‐TCTTCTTCAGTCGCTCCAGGTCTT‐3

### Western blot

Total protein was extracted from colon tumours and their adjacent normal tissues using RIPA lysis buffer with inhibitor phenylmethanesulfonyl fluoride (PMSF), and then the concentration was measured with BCA protein assay kit (Beyotime Biotechnology, Beijing, China). Western blots were performed with specific antibodies to detect the corresponding proteins. After incubation at 4°C overnight, the blot was washed three times with 0.05% Tween‐20 TBS (TBST) and then incubated with 1:5000 diluted goat anti‐rabbit or mouse IgG conjugated with HRP for 2 hrs at room temperature. After additional washing with TBST, the target proteins on the blot membrane were visualized using the ECL system. The optical density (OD) of each band was analysed using the Image‐J software (National Institutes of Health, USA).

### Cell proliferation assays

Cells were incubated for 24 hrs at 37°C after the cells were trypsinized and seeded into 96‐well plates (1 × 10^3^ per well). Then 10 μl Cell Counting Kit‐8 (CCK‐8; Dojindo Laboratories, Japan) was added and mixed into each well and incubated for 1 hr at 37°C, and then absorbance was measured at 450 nm. Three dependent experiments were repeated. Data were presented as the mean ± S.D.

### Colony formation assay

Two hundred cells per well were trypsinized and cultured on six‐well plates in medium with 10% FBS containing 5% CO_2_ for 2 weeks. The cells were then fixed with methyl alcohol for 30 min. and stained with 1% crystal violet for 10 min. Colonies of more than 50 cells were counted. All experiments were performed in triplicate. Data were presented as the mean ± S.D.

### Cell cycle analysis

The cell cycle of CT26 and Sw620 cells was analysed by flow cytometry using BD FACSAria Cell Sorter (Becton Dickinson, Franklin Lakes, NJ, USA). Scramble group and CD155 knockdown groups were seeded onto six‐well dishes for 24 hrs. After incubation, the cells were trypsinized and collected by centrifugation and fixed with 70% ethanol. Samples were treated with 5 μl RNase A (10 μg/ml) for 1 h at room temperature, stained with 10 μl propidium iodide (10 μg/ml) for 30 min. at 4°C and analysed using the FACSCalibur flow cytometer (BD Company, New Jersey, USA).

### Tumour cell migration and invasion assays

For migration assay, 5 × 10^4^ cells were allowed to migrate from upper to lower chamber for 14 hrs. Then the migration was stopped. All cells were fixed with cold 4% paraformaldehyde (PFA) for 30 min. and stained with 0.1% crystal violet for 30 min., followed by washed three times with PBS and mounted on glass slides. Ten different fields of each membrane were selected randomly for the images capture. The numbers of migration cells were counted to determine the index of cell migration. For invasion assay, 1 × 10^5^ cells were allowed to migrate from upper to lower chamber. After 24 hrs’ migration, the treatment of chambers was carried out as above. All experiments were performed in triplicate. Data were presented as the mean ± S.D.

### Flow cytometric (FCM) analysis

The apoptotic status was analysed by an Annexin V‐PE/7‐AAD Apoptosis Kit (559763; BD Biosciences, New Jersey, USA). Cells were stained and evaluated for apoptosis by flow cytometry according to the manufacturer's protocol. The number of apoptotic cells was analysed by flow cytometry (BD Biosciences, New Jersey, USA).

### Tumour xenotransplantation

CD155 knockdown cells were trypsinized and then suspended with serum‐free medium. 200 μl (2 × 10^6^ cells) suspension was subcutaneously injected into 5‐week‐old nude athymic female mice or BALB/C female mice (Weitong Lihua, Beijing, China). Scramble CT26 cells were used as negative control. Subcutaneous tumour development was monitored by palpation once a week, and tumour sizes were recorded. The tumour volume was calculated by the formula of *V* = ab^2^/2(cm^3^). The mice were housed and maintained in specific pathogen‐free conditions in facilities approved by the Animal Department of China Medical University. All procedures were carried out under approved Institutional Animal Care and Use Committee protocols. Humane care of the mice was thoroughly considered.

### Statistical analysis

All experiments were obtained at least in three replicates. In addition, assays producing quantitative data were run in triplicate. SPSS 19.0 software (SPSS Company, USA) was employed. Statistical significance was determined by one‐way anova or two‐tailed Student′s *t*‐test. The association of protein expression with clinicopathological features was analysed by the Pearson chi‐square test. *P* < 0.05 was considered statistically significant.

## Results

### CD155 is overexpressed in colorectal cancers

**Figure 1:**
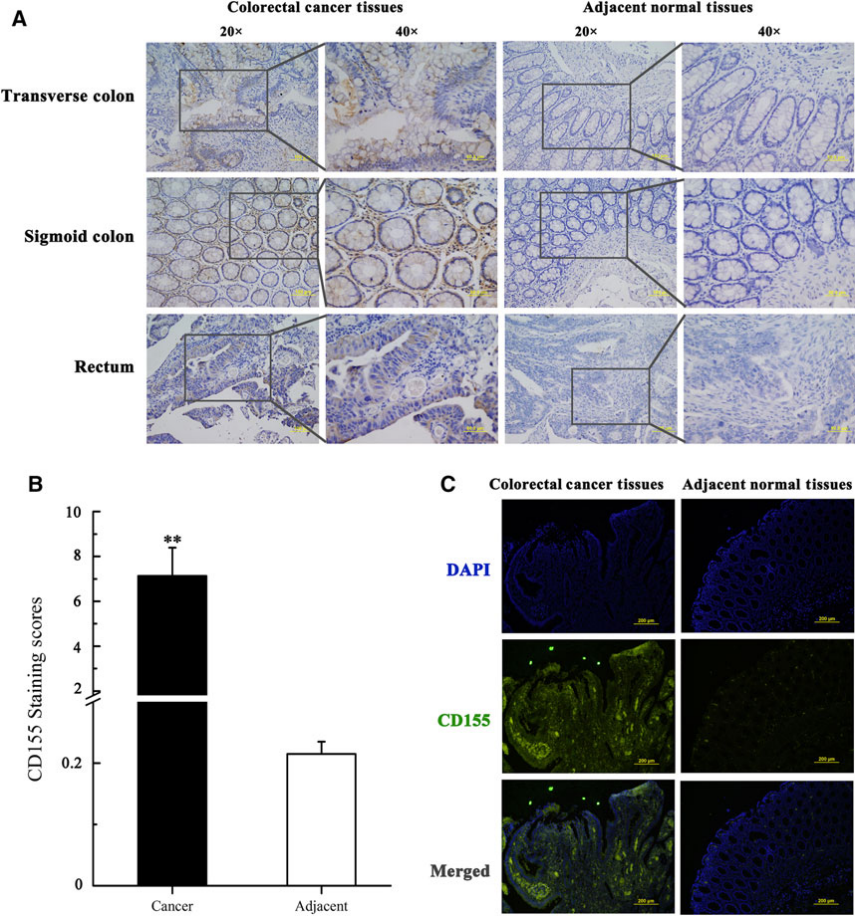
CD155 is up‐regulated in colorectal cancer tissues. (**A**) Expression levels of CD155 protein in 97 paired CRC tissues, and paired adjacent normal tissues were determined by immunohistochemistry (IHC). The data were shown with different magnification (20× and 40×). (**B**) IHC expression of CD155 quantified by expression score (0–10) in CRC tissues and adjacent normal tissues. The levels of CD155 in CRC tissues are significantly higher than those in adjacent tissues. **P<0.01. (**C**) Representative IF histochemistry analysis of CD155 protein in CRC tissues and adjacent normal tissues.

**Figure 2:**
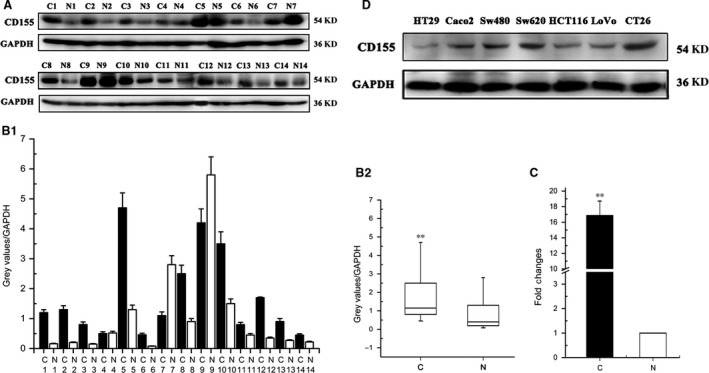
CD155 expression in CRC tissues and cell lines. (**A**) Expression levels of CD155 protein in 14 paired CRC tissues were determined by Western blot. (C: Cancer; N: Normal) (**B1**,** B2**) Relative expression density of CD155 was quantified. GAPDH was used as a loading control. CD155 expressions were higher in CRC tissues than those in adjacent normal tissues (**P < 0.01). (**C**) Relative expression levels of CD155 mRNA in CRC tissues and paired adjacent normal tissues (*n* = 14) by qPCR. The levels of CD155 in CRC tissues are significantly higher than those in adjacent normal tissues (**P < 0.01). (**D**) Expression levels of CD155 were detected in different colon cancer cell lines (HT29, Caco2, Sw480, Sw620, HCT116, LoVo and CT26) by Western blot analysis.

CD155 expression was detected in 97 colorectal cancer samples with matched adjacent normal tissues by immunohistochemistry. It was found that CD155 was positively expressed in 84 (86.6%) cancer specimens and 10 (10.3%) normal tissues, respectively (Table 3, Fig. 1A and B). The results showed that CD155 expression was up‐regulated in colorectal cancers. CD155 overexpression was confirmed by IF histochemistry, Western blot and real‐time PCR (Figs 1C and 2A–C). CD155 expression was also found in six human colon cancer cell lines and mouse colon cancer cell line CT26 (Fig. 2D). Clinicopathological analysis revealed that CD155 expression was correlated with Duke's Degree, AJCC stage and metastasis, indicating that CD155 overexpression is implicated in colorectal cancer progression (Table 1).

**Table 3:** CD155 expression in CRC tissues compared with adjacent normal tissue

CD155 Expression	Tumour tissue (*n* = 97)	Adjacent tissue (*n* = 97)	*P*‐value
Negative	14	87	0.000
Positive	83	10	

*P* < 0.05 indicates a significant association between the variables.

Statistical analysis was carried out with Pearson chi‐square test.

### CD155 knockdown suppresses colon cancer cell proliferation

**Figure 3:**
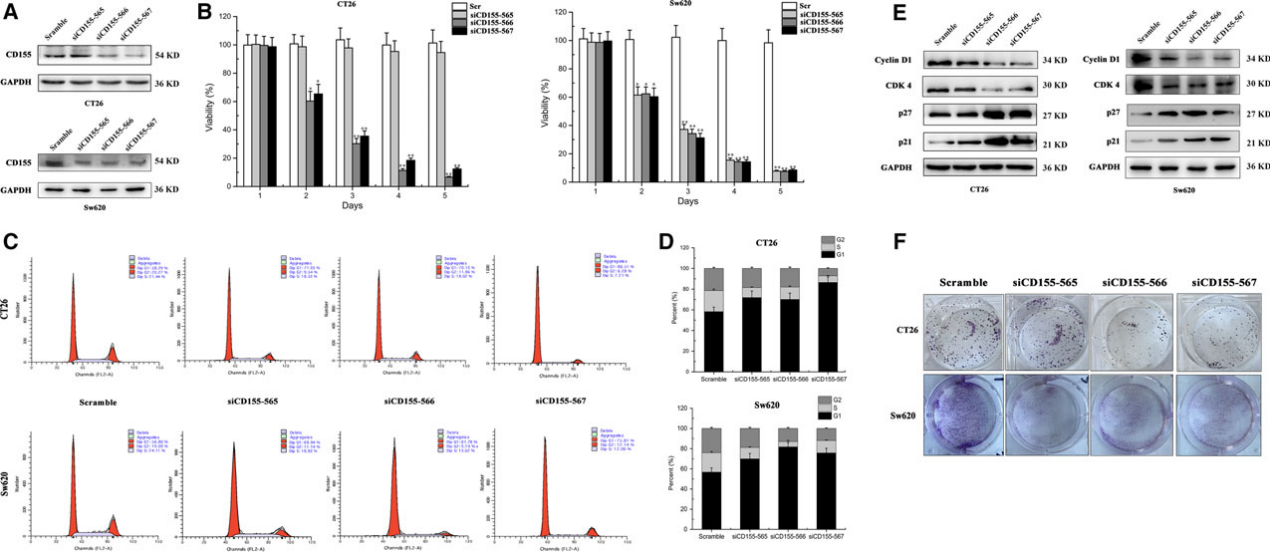
Effect of CD155 gene silencing on the growth of CT26 and Sw620 cell lines. (**A**) Western blot analysis was performed to assess CD155 knockdown efficiency in CT26 and Sw620 cells. (**B**) CCK‐8 assay was performed to determine the viability of CT26 and Sw620 cells transfected with scramble and siRNA CD155 (565, 566 and 567). The data represent the mean ± S.D. from three independent experiments (*P < 0.05, **P < 0.01). (**C**) Cells were trypsinized, and cell cycle was analysed with FACS. (**D**) Per cent of G1, S and G2 phases was analysed in different cell lines. (**E**) Western blot confirmed that Cyclin D1 and CDK4 were decreased, while p21 and p27 were up‐regulated in CD155 knockdown cells. (**F**) A colony formation assay was carried to determine the effect of CD155 knockdown on CT26 and Sw620 cells. The colonies were counted and captured.

To evaluate the role of CD155 in colon cancer cell proliferation, CT26 and Sw620 cells were transfected with CD155 shRNA lentiviruses (Fig. 3A). CCK‐8 detection showed that viability of CT26 and Sw620 cells was significantly inhibited by CD155 knockdown (Fig. 3B). Moreover, flow cytometry analysis indicated that CD155 knockdown led to cell cycle arrest in G1 phase (Fig. 3C and D). The underlying mechanisms were also investigated. Western blot detection showed that CD155 knockdown down‐regulated CyclinD1 and CDK4 expression, whereas it up‐regulated p27 and p21 expression (Fig. 3E). Consistent with CCK‐8 detection and cell cycle analysis, colony formation assay revealed that CD155 knockdown weakened colony formation ability of CT26 and Sw620 cells (Fig. 3F). These results suggest that CD155 overexpression may promote colon cancer cell proliferation.

### CD155 knockdown inhibits colon cancer cell migration and invasion

**Figure 4:**
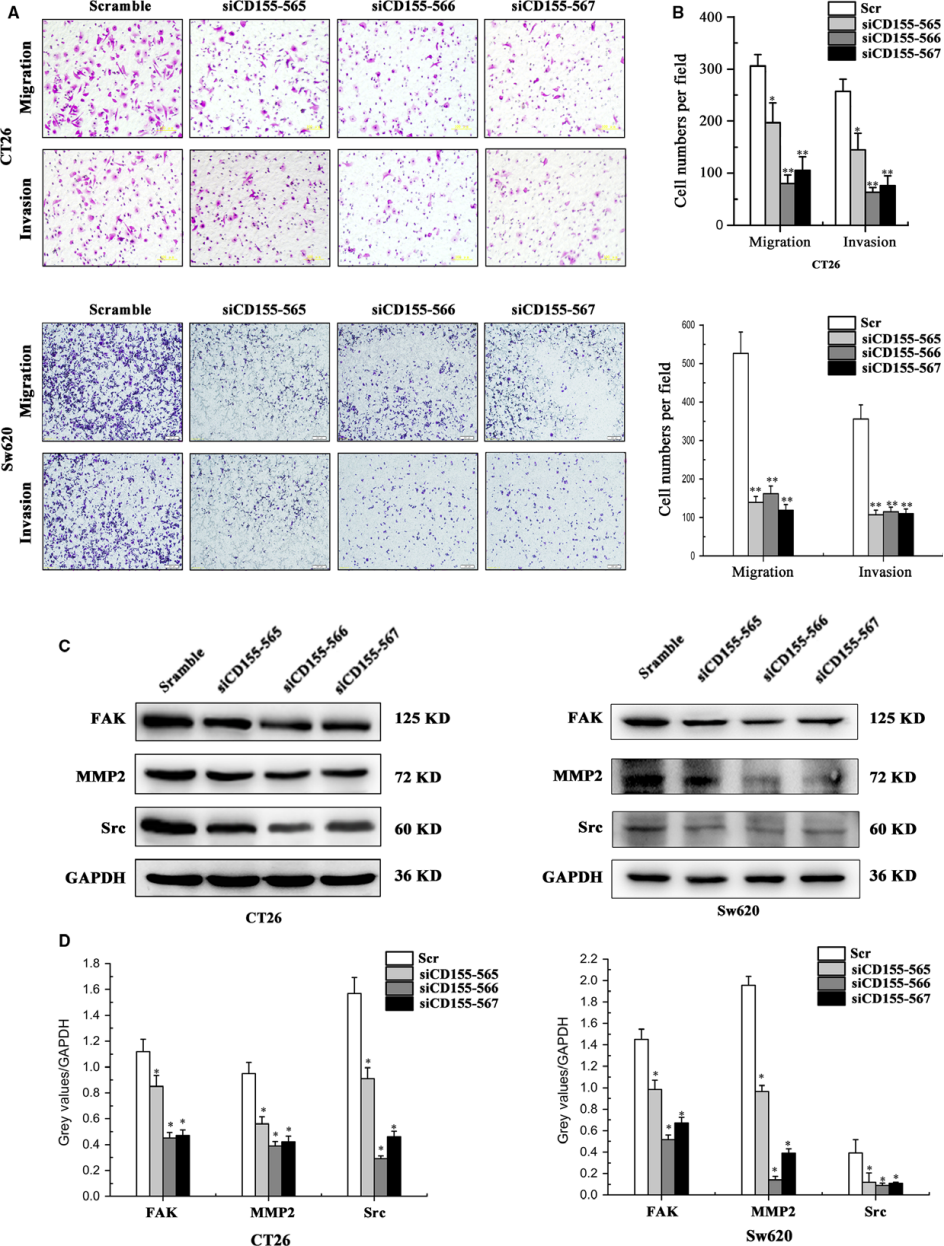
CD155 knockdown inhibited CT26 and Sw620 cells migration and invasion. (**A**) Transwell migration and Matrigel invasion assays were carried out to prove that siCD155 inhibited cell migration and invasion ability compared with the scramble group. (**B**) Quantitative results of migration and invasion assays. The differences in cell migration and invasion were statistically significant. The data represent the mean ± S.D. from three independent experiments (*P < 0.05, **P < 0.01). (**C**) Western blot confirmed that FAK, MMP2 and Src were depressed in CD155 knockdown cells compared with scramble group. (**D**) Relative expression density of CD155 was quantified. GAPDH was used as a loading control. (*P < 0.05).

The role of CD155 in colon cancer cell migration and invasion was assessed by transwell assay. It was found that CD155 knockdown significantly inhibited migration and invasion of CT26 and Sw620 cells (Fig. 4A and B). Further studies indicated that expression of FAK, Src and MMP‐2 decreased significantly in CT26 and Sw620 cells after transfection with CD155 shRNA lentiviruses, suggesting that these molecules may be responsible for CD155‐induced colon cancer cell migration and invasion (Fig. 4C and D).

### CD155 knockdown promotes colon cancer cell apoptosis

**Figure 5:**
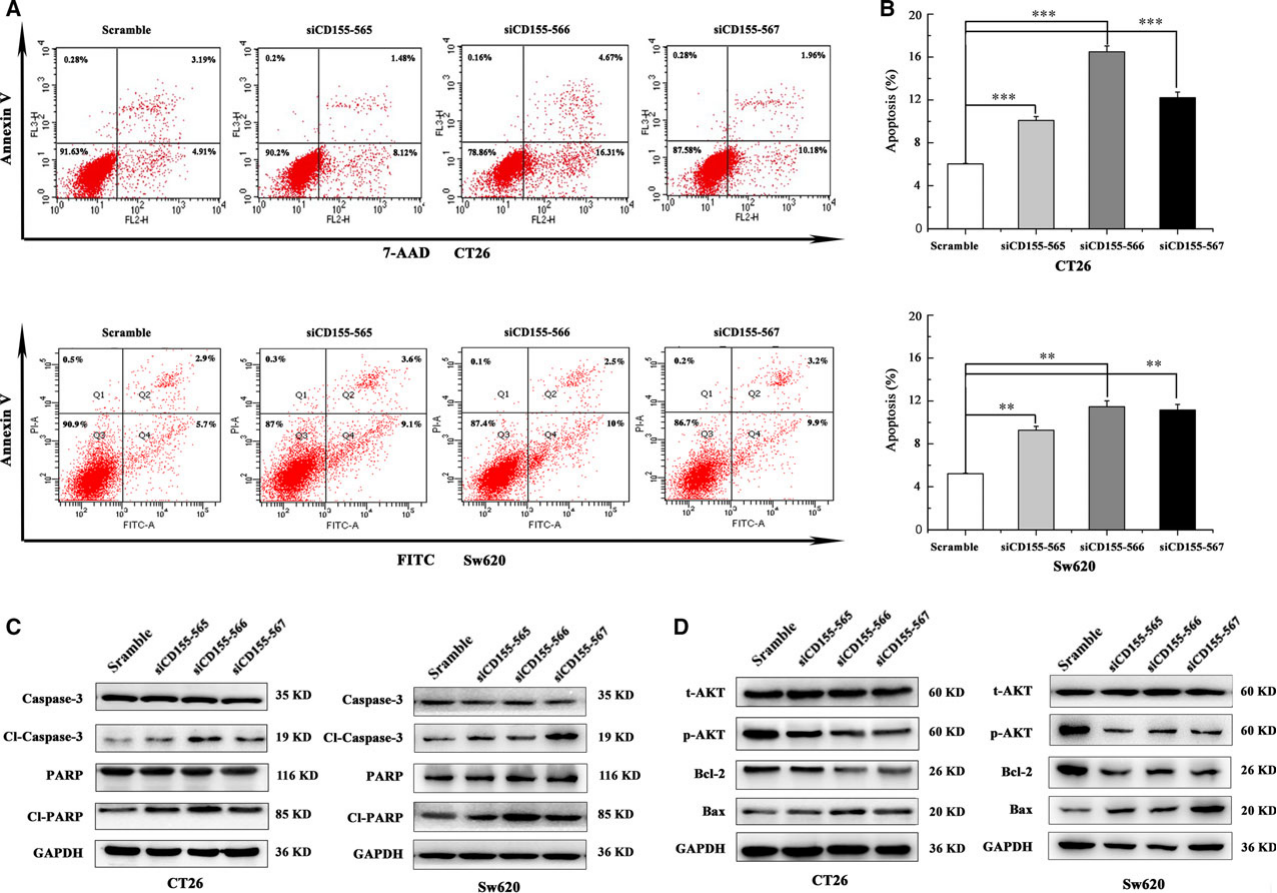
CD155 knockdown improved CT26 and Sw620 cells apoptosis. (**A**) CD155 knockdown significantly increase the apoptosis rate of CT26 and Sw620 cells. (**B**) Compared with scramble group, the differences among the knockdown groups were statistically significant. The data represent the mean ± S.D. from three independent experiments (**P < 0.01, ***P < 0.001). (**C**,** D**) Western blot assay was carried out to detect the expression levels of caspase‐3, cleaved caspase‐3, PARP, cleaved PARP and t/p‐AKT, Bcl‐2, Bax in CT26 and Sw620 cells. GAPDH was used as the loading control.

CD155 has been demonstrated to regulate the functions of some growth factors; therefore, we speculated that CD155 might be related to cancer cell survival, and then explored whether CD155 had a role in colon cancer cell apoptosis. Just as expected, CD155 knockdown remarkably stimulated apoptosis of CT26 and Sw620 cells (Fig. 5A and B). Further studies indicated that expression of cleaved caspase‐3 (Cl‐caspase‐3) and cleaved PARP (Cl‐PARP) was increased after CD155 shRNA lentivirus transfection (Fig. 5C). Moreover, CD155 knockdown up‐regulated pro‐apoptotic Bax, whereas it down‐regulated anti‐apoptotic Bcl‐2 (Fig. 5C). PI3K/Akt pathway mediates the intracellular signalling of some growth factors and plays an anti‐apoptotic role in tumour cells. This study revealed that CD155 knockdown reduced the expression of phosphorylated Akt, suggesting PI3K/Akt pathway may be responsible for CD155‐induced colon cancer cell survival (Fig. 5D).

### CD155 knockdown suppresses colon cancer cell growth in xenograft mice

**Figure 6:**
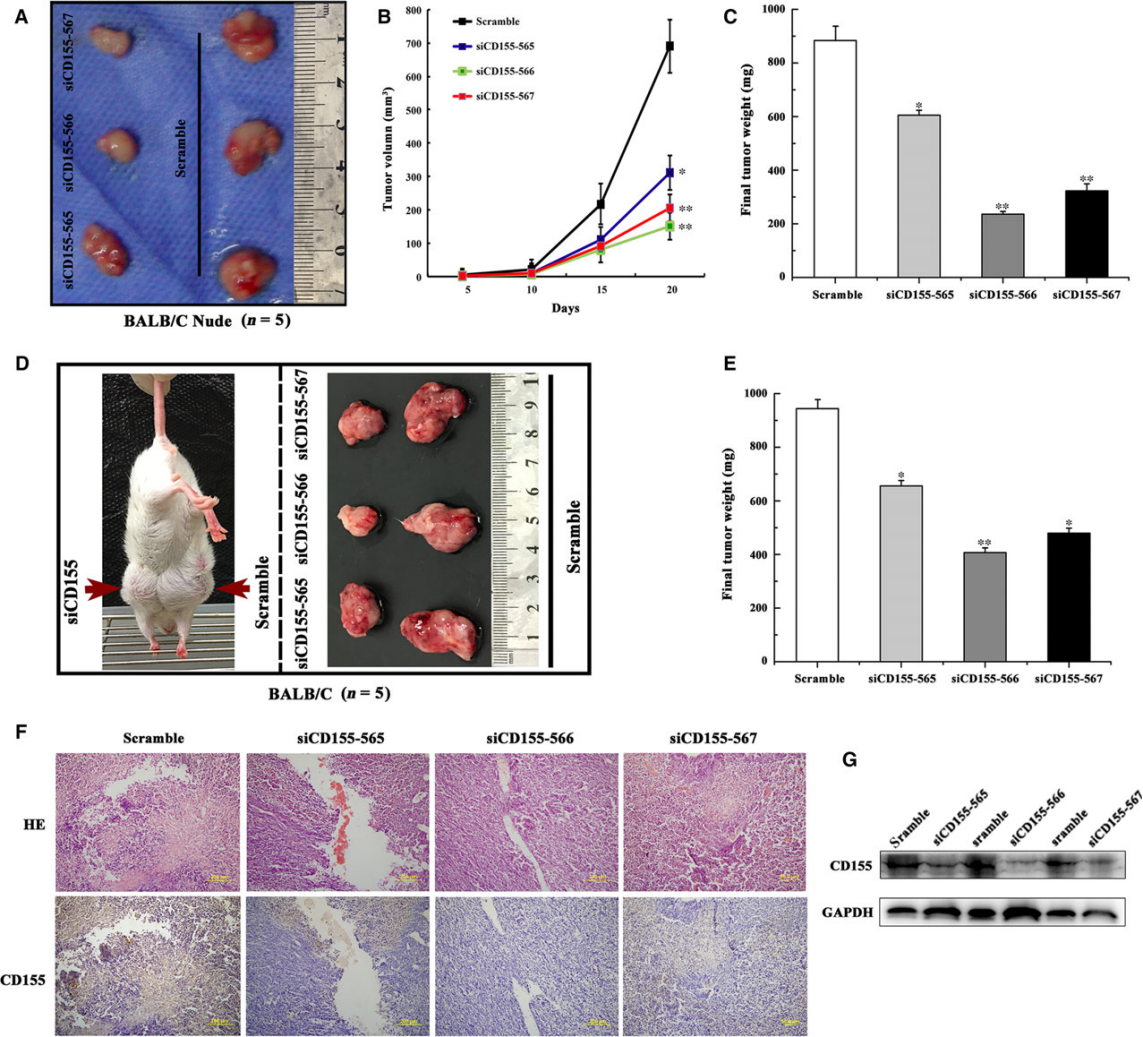
CD155 knockdown suppressed CT26 cells tumorigenesis *in vivo*. (**A**) Tumorigenesis assay by subcutaneous injection of CT26/Scr and CT26/siCD155‐(565, 566 and 567) cells in nude mice (*n* = 5/group). (**B**) The tumour growth volume was measured every 5 days. The results are presented as the means ± S.D. (*n* = 5/group). (**C**,** E**) Final tumour weights were measured after the tumours were surgically dissected (*P < 0.05, **P < 0.01). (**D**) Tumorigenesis assay by subcutaneous injection of CT26/Scr and CT26/siCD155‐(565, 566 and 567) cells in BALB/C mice (*n* = 5/group). (**F**) Representative images of haematoxylin and eosin staining and IHC staining of tissue sections from tumour bearing mice that were injected with CT26/Scr and CT26/siCD155‐(565, 566 and 567) cells. IHC specimens were stained with antibodies specific CD155. (**G**) Western blot analysis was carried out to detect expression level of CD155 in different tumour tissues.

To study the role of CD155 on tumour growth *in vivo*, CT26 cells with or without CD155 knockdown were inoculated into nude mice for 3 weeks. The results showed that both tumour volume and tumour weight in CD155 knockdown group were significantly smaller than those in control group (Fig. 6A–C). Moreover, CT26 cells with or without CD155 knockdown were inoculated into wild‐type BALB/C mice, and similar results were observed (Fig. 6D and E). Immunohistochemistry and Western blot detection showed that CD155 expression reduced in CD155 shRNA tumours compared to controls (Fig. 6F and G). These results indicate that CD155 maintains colon cancer cell growth *in vivo*.

## Discussion

Our immunohistochemistry study clearly demonstrated that CD155 protein was overexpressed in colorectal cancer tissues, consistent with real‐time study in which *CD155* mRNA was also up‐regulated, compared with adjacent normal tissues. Like NIH3T3 cells 5, CD155 seemed to maintain colon cancer cell proliferation, verified by that CD155 down‐regulation inhibited colon cancer cell proliferation and colony formation. Further exploration revealed some molecular mechanisms underlying CD155‐maintained cell proliferation. CD155 knockdown blocked the expression of cyclin and CDK, but induced the expression of CDK inhibitors. These results suggest that overexpression of CD155 is associated with colon cancer growth.

As one of nectin‐like molecules, CD155 interacts with adhesion molecule nectin to regulate cell movement. Actually, by *in vitro* transwell analysis, our study showed that CD155 down‐regulation significantly impaired colon cancer invasion and migration. This observation was consistent with clinicopathological analysis of colorectal cancer samples, which indicated that CD155 expression was correlated with Duke's Degree stage and metastasis. These studies suggest that CD155 attributes to tumour cell invasion and migration and then colon cancer metastasis. Subsequent study revealed that CD155 knockdown reduced the expression of some molecules related to cell invasion and migration, such as FAK, Src and MMP‐2.

It has been unexplored whether CD155 plays a role in cancer cell apoptosis. As CD155 has been shown to be involved in the function of some growth factors, it is reasonable to speculate that CD155 is related to cell survival. Indeed, CD155 knockdown notably induced colon cancer cell apoptosis, with increased expression of cleaved caspase‐3 and cleaved PARP. Apoptosis induced by CD155 down‐regulation seemed to be related to the unbalance of anti‐apoptotic and pro‐apoptotic gene products. Moreover, CD155 knockdown inhibited the phosphorylation of Akt, an important molecule in cell apoptosis, suggesting that PI3K/Akt pathway may be involved in CD155‐related cancer cell survival. Together, these findings indicate that CD155 is an anti‐apoptotic molecule in human colon cancer.

A substantial of evidence indicates that CD155 has immunoregulatory function. Interactions of CD155 with its stimulatory receptor CD226 (DNAX accessory molecule‐1, DNAM‐1) could induce NK and T cell‐mediated cytotoxicity 15, 16. Actually, *in vitro* studies showed that CD155‐positive tumour cells were susceptible to NK‐mediated lysis, whereas CD155‐negative tumour cells were resistant to lysis 17, 18. However, several studies indicated that CD155 derived from tumour cells and DCs down‐regulated CD226 expression 19, 20, 21.

In summary, the present study investigated the role of CD155 overexpression in colorectal cancer. The results showed that CD155 knockdown impaired colon cancer cell proliferation, invasion and migration, suggesting that CD155 overexpression is involved in colon cancer progression. Moreover, our study for the first time revealed that CD155 is an anti‐apoptotic factor in this cancer. Whether does CD155 play anti‐apoptotic role in other human cancers? What is the role of CD155 in immunoregulation of colorectal cancer? These questions need to be further studied.

## Conflict of interest

There is no conflict of interest.
